# The Neuropeptides Vasoactive Intestinal Peptide and Pituitary Adenylate Cyclase-Activating Polypeptide Control HIV-1 Infection in Macrophages Through Activation of Protein Kinases A and C

**DOI:** 10.3389/fimmu.2018.01336

**Published:** 2018-06-12

**Authors:** Jairo R. Temerozo, Suwellen S. D. de Azevedo, Daniella B. R. Insuela, Rhaíssa C. Vieira, Pedro L. C. Ferreira, Vinícius F. Carvalho, Gonzalo Bello, Dumith Chequer Bou-Habib

**Affiliations:** ^1^Laboratory on Thymus Research, Oswaldo Cruz Institute/Fiocruz, Rio de Janeiro, Brazil; ^2^National Institute of Science and Technology on Neuroimmunomodulation, Oswaldo Cruz Institute/Fiocruz, Rio de Janeiro, Brazil; ^3^Laboratory of AIDS and Molecular Immunology, Oswaldo Cruz Institute/Fiocruz, Rio de Janeiro, Brazil; ^4^Laboratory of Inflammation, Oswaldo Cruz Institute/Fiocruz, Rio de Janeiro, Brazil

**Keywords:** HIV-1, vasoactive intestinal peptide, pituitary adenylate cyclase-activating polypeptide, neuropeptides, macrophages, protein kinase C, protein kinase A

## Abstract

Vasoactive intestinal peptide (VIP) and pituitary adenylate cyclase-activating polypeptide (PACAP) are highly similar neuropeptides present in several tissues, endowed with immunoregulatory functions and other systemic effects. We previously reported that both neuropeptides reduce viral production in HIV-1-infected primary macrophages, with the participation of β-chemokines and IL-10, and now we describe molecular mechanisms engaged in this activity. Macrophages exposed to VIP or PACAP before HIV-1 infection showed resistance to viral replication, comparable to that observed when the cells were treated after infection. Also, multiple treatments with a suboptimal dose of VIP or PACAP after macrophage infection resulted in a decline of virus production similar to the inhibition promoted by a single exposure to the optimal inhibitory concentration. Cellular signaling pathways involving cAMP production and activation of protein kinases A and C were critical components of the VIP and PACAP anti-HIV-1 effects. Analysis of the transcription factors and the transcriptional/cell cycle regulators showed that VIP and PACAP induced cAMP response element-binding protein activation, inhibited NF-kB, and reduced Cyclin D1 levels in HIV-1-infected cells. Remarkably, VIP and PACAP promoted G-to-A mutations in the HIV-1 provirus, matching those derived from the activity of the APOBEC family of viral restriction factors, and reduced viral infectivity. In conclusion, our findings strengthen the antiretroviral potential of VIP and PACAP and point to new therapeutic approaches to control the progression of HIV-1 infection.

## Introduction

The neuropeptides vasoactive intestinal peptide and pituitary adenylate cyclase-activating polypeptide (VIP and PACAP, respectively) are members of the secretin/glucagon family of peptides and are distributed systemically; VIP and PACAP act through three G-protein coupled receptors that are expressed in several cell types, namely, VPAC1, VPAC2, and PAC1 (1, 2). Both neuropeptides have several regulatory functions in the neuro-immune-endocrine system, and they mainly control cytokine production, cell activation, and differentiation (1, 2). VIP and PACAP signaling depends on which of their receptors are activated and includes complex pathways. The main proteins involved in the initial stages of the cell signaling triggered by VIP and PACAP are the protein kinases A (PKA) and C (PKC), which respond to these neuropeptides by modulating transcription factors and cell cycle proteins (3, 4). Both neuropeptides are considered to be potential targets of therapeutic approaches for autoimmune disorders and chronic inflammatory illnesses due to their remarkable anti-inflammatory activities (5, 6).

Macrophages play a critical role in the pathogenesis of HIV-1 infection due to their resistance to the cytopathic effects that are secondary to viral replication and to their ability to continuously produce virus particles. These cells are important reservoirs of HIV-1 because of their ability to escape from immune system surveillance and produce virions when activated by stimuli derived from the virus itself and the tissue microenvironment (7–9). HIV-1 infection may become latent in macrophages, a condition that allows the formation, maintenance, and cycling of viral reservoirs and that constitutes a major obstacle to the therapeutic control of HIV-1 infection (7–9). During viral transcription, the HIV-1 transactivation protein Tat recruits the Cyclin/Cyclin-dependent kinase (CDK) family, eliciting productive transcription of the HIV-1 genome, along with the participation of the transcription factors NF-κB and NFAT (10–13). The absence of these complexes causes incomplete or abortive transcription of the viral genome (13, 14). HIV-1 production is also controlled by cellular factors that restrict viral replication, such as the interferon-stimulated protein APOBEC3G. APOBEC3G induces mutations in the viral genome, affecting the replicative capacity of the nascent virus (15, 16). VIP and PACAP are known to prevent the activity of some Cyclin/CDK complexes by increasing the production of their inhibitors (17–19) and are also capable of regulating the activity of NF-kB and NFAT (20, 21). In addition, PKA and PKC phosphorylate and modulate the activity and transcription of APOBEC3G and other members of the APOBEC family, thus regulating the insertion of mutations into the HIV-1 genome (22–24). Therefore, VIP and PACAP may modulate the HIV-1 replication by controlling the availability of essential components for the establishment of a productive infection.

Some authors have shown that activation of the VIP/PACAP receptors VPAC1 and VPAC2 by specific ligands alters viral replication in peripheral blood mononuclear cells (PBMCs) and in lymphocytic lineages (25, 26), and we also reported that both neuropeptides inhibit HIV-1 replication in macrophages through production of β-chemokines and IL-10 (27). We also found that the ability of VIP and PACAP to inhibit HIV-1 replication is dependent on activation of VPAC2 and PAC1, whereas specific VPAC1 activation increases HIV-1 production in these cells. Now, we describe molecular mechanisms by which both neuropeptides reduce HIV-1 production, and show that components associated with proviral transcription are recruited by VIP and PACAP to control HIV-1 replication in primary macrophages.

## Materials and Methods

### Primary Cells and Cell Lines

Human monocyte-derived macrophages were obtained from PBMCs that had been isolated by density gradient centrifugation (Ficoll-Paque Premium, GE Healthcare Life Sciences) from buffy-coat preparations of blood from healthy donors, through adherence onto plastic plates, as described (28). Briefly, 10^6^ PBMCs were plated onto 96-well plates (Costar) in DMEM low-glucose (DMEM; LGC Bio) containing 10% human serum (Millipore) and penicillin–streptomycin (Gibco). Cells were maintained at 37°C in 5% CO_2_ for 7–8 days for monocyte differentiation into macrophages. Non-adherent cells were washed out (three washes with PBS), and the remaining macrophage layer was maintained in DMEM with 5% human serum. Macrophage purity was >90%, as determined by flow cytometry analysis using anti-CD3 (BD Bioscience) and anti-CD68 (BD Bioscience) monoclonal antibodies. For some assays (see below), macrophages were prepared in 25 cm^2^ plastic culture flask, following the same protocol, but dispensing 4 × 10^7^ PBMCs/5 mL medium/flask, or in six-well plates with 10^7^ PBMCs/3 mL. The approximate number of macrophages obtained in each approach is indicated in the respective technical procedure. The human monocytic leukemia cell line THP-1 (ATCC: TIB202TM) was maintained in DMEM with low-glucose (LGC Bio) supplemented with 10% heat-inactivated fetal calf serum (Cultilab) and penicillin–streptomycin and differentiated into macrophages by treating them with 40 ng/mL of PMA for 3 days. Then, the cells were washed three times with PBS and incubated with fresh medium for an additional 3 days. TZM-bl cells (obtained through the AIDS Research and Reference Reagent Program, NIH, MD, USA; Dr. John C. Kappes, Dr. Xiaoyun Wu, and Tranzyme Inc.) were maintained with DMEM low-glucose with 10% heat-inactivated fetal calf serum and penicillin–streptomycin.

### HIV-1 Isolates, Reagents, and ELISA Kits

Assays of macrophage infection were performed with the CCR5-dependent isolate HIV-1 Ba-L (donated by the AIDS Research and Reference Reagent Program, NIH, MD, USA), which was expanded in phytohemagglutinin-activated PBMCs from healthy donors, as described elsewhere (29). Recombinant human VIP and PACAP and the pharmacological inhibitors (PKA: H89; PKC: Go6383; and the PKA/PKC/PKG inhibitor H7) were purchased from Tocris, and the pertussis toxin (PTX) from Sigma-Aldrich. The HIV-1 p24 ELISA kits were acquired from Sino Biological, and MIP-1α and IL-10 ELISA kits were purchased from R&D Systems and eBioscience, respectively.

### HIV-1 Infection and Evaluation of Neuropeptide Effects on HIV-1 Replication

Macrophages (5 × 10^4^/200 μL/well, 96-well plates) were infected with HIV-1 by exposing them overnight to viral suspensions containing 10 ng/mL of p24 antigen, as we have described (27). Then, non-internalized viruses were removed by washing (three times with PBS), and cell monolayers were replenished with fresh medium. HIV-1 replication was quantified in cell culture supernatants after 10–12 days of infection by a commercial ELISA kit (Sino Biological), according to manufacturer’s instructions. HIV-1-infected macrophages were treated either with VIP or PACAP immediately after cell infection, or, in some assays, before infection, and maintained during culture. Cells were maintained in culture for different time-points, and HIV-1 replication was measured as described above. To measure the impact of pharmacological inhibitors on the neuropeptide effects on HIV-1 replication, HIV-1-infected macrophages were treated with the appropriate inhibitor for 30 min and, then, cells were washed and the neuropeptides added.

### cAMP, NF-kB, cAMP Response Element-Binding Protein (CREB), Cyclin D1, MIP-1α, and IL-10 Measurement Assays

For cAMP quantification, macrophages (5 × 10^4^/200 μL/well, 96-well plates) were treated with 500 nM of IBMX (a competitive nonselective phosphodiesterase inhibitor, to avoid cAMP degradation) and, after 15 min, with VIP or PACAP (10 nM), for different time-points. Culture supernatants were removed, cells were lysed with 0.1 M HCl, and intracellular cAMP levels were determined by ELISA according to the manufacturer’s instructions (Cayman Chemical). For NF-kB, CREB, and Cyclin D1 measurement analyzes, macrophages infected or not with HIV-1 were treated with VIP or PACAP (10 nM), following different protocol settings (see Results), and thus used for ELISA assays performed according to manufacturer’s instructions: NFkB p65 (Total/Phospho) InstantOne™, CREB (Total/Phospho) Multispecies InstantOne™ ELISA Kits (Thermo Fisher), and PathScan^®^ Total Cyclin D1 Sandwich ELISA Kit (Cell Signaling). For MIP-1α and IL-10 quantification, infected macrophages were treated with VIP or PACAP and, 48 h later, supernatants were collected and analyzed with specific ELISA kits.

### Immunoblotting for Detection of PKA and PKC Activation

Macrophages (1.5 × 10^6^/4 mL/flask, 25 cm^2^ flasks) were treated with VIP or PACAP for different time-points and then proteins were extracted using RIPA buffer (Thermo Fischer) with Protease Inhibitor Cocktail Set III and Phosphatase Inhibitor Cocktail Set II (Merck). The protein concentration was quantified by Qubit 2.0 Protein Assay Kit (Thermo Fisher). Equal amounts of sample protein were separated by SDS-PAGE using polyacrylamide gels, and proteins were transferred to nitrocellulose membranes (GE Healthcare). Nonspecific binding was blocked with 5% (w/v) skimmed milk powder in TTBS (Tween 20 tris-buffered saline) for 1 h, followed by incubation with rabbit polyclonal anti-phospo-PKA (1:1,000; sc-32968, Santa Cruz Biotechnology), mouse monoclonal anti-PKA (1:1,000; sc-390548, Santa Cruz Biotechnology), rabbit polyclonal anti-phospho-PKC (1:1,000; ab23513, Abcam), mouse monoclonal anti-PKC (1:1,000; ab23511, Abcam), or mouse monoclonal anti-β-actin antibody (1:3,000; ab8226, Abcam), overnight at 4°C. Then, membranes were washed with TTBS and incubated with HRP-conjugated secondary antibodies (1:1,000; HAF007 or HAF008, R&D Systems) or IRDye secondary antibodies (1:15,000; 925–32,210, 925–32,211, LI-COR Corporate) for 1 h at room temperature. The membranes were washed in TTBS and protein expression was detected using enhanced chemiluminescence (SuperSignal West Dura, Thermo Fisher) or fluorescence using the Odyssey Image System (LI-COR Corporate). Bands intensity was quantified by densitometry (Image-Pro^®^ Plus Media Cybernetics).

### Luciferase Assay

To investigate the NF-kB-dependent transcriptional activity, THP-1 cells (4 × 10^4^ cells/well, 96-well plates) were transfected with 100 ng of p6kB-LUC (kindly provided by Dr. Ulisses G. Lopes, UFRJ, Brazil) and 2 ng pRL-CMV (Promega), using PolyFect Transfection Reagent (Quiagen). Transfected cells were treated with TNF-α (10 ng/mL) and, 1 h later, exposed to VIP or PACAP (10 nM). After 24 h, cells were washed (three times with PBS), lysed according to Dual Luciferase System protocol (Promega), and the NF-kB activation was analyzed in a SpectraMax M3 Luminometer (Molecular Devices).

### HIV-1 LTR Sequence Analyses

HIV-1-infected macrophages (10^6^/3 mL/well, six-well plates) were treated with VIP (10 nM), PACAP (10 nM), or IFN-α (10^3^ U/mL) and, after 12 days, DNA was extracted using a QIAamp DNA kit (Qiagen Inc.), and quantified using a Nanodrop 1000 system (Thermo Fisher). The 5′LTR region was amplified by a nested-PCR protocol and directly sequenced as described (30). Chromatograms were assembled into contigs using the SeqMan v7.0 software (DNASTAR Inc.). Nucleotide sequences were aligned using ClustalW implemented in MEGA 7 program (31) and then edited, yielding an alignment covering positions 57–580 relative to the HXB2 reference genome. Neighbor-Joining phylogenetic trees were reconstructed under the Tamura–Nei nucleotide substitution model (32) using the Mega v7 program. Phylogenetic confidence was assessed by bootstrap with 1,000 replicates, and pairwise genetic distances were estimated under the Tamura–Nei nucleotide substitution model using the Mega v7 program.

### Replication Fitness Assays

HIV-1-infected macrophages were treated with VIP (10 nM), PACAP (10 nM) or IFN-α (10^3^ U/mL) and, after 12 days, culture supernatants were collected, centrifuged at 3,000 × *g*, filtered using 45 μm pore size, and virions were concentrated using Centricon filter devices with YM-100 membranes (Millipore). These supernatants were obtained from the HIV-1-infected macrophages used for LTR sequencing experiments. Next, virus amount was quantified (by p24 ELISA, as above) and TZM-bl cells were infected with 5 ng/mL of normalized virus suspensions in the presence of DEAE-Dextran (15 μg/mL). Luciferase activity was assessed with Bright-Glo reagent (Promega) 48 h after infection.

## Results

### VIP and PACAP Retain Their Anti-HIV-1 Effects Under Diverse Addition Protocols to Macrophages

**Figure 1:**
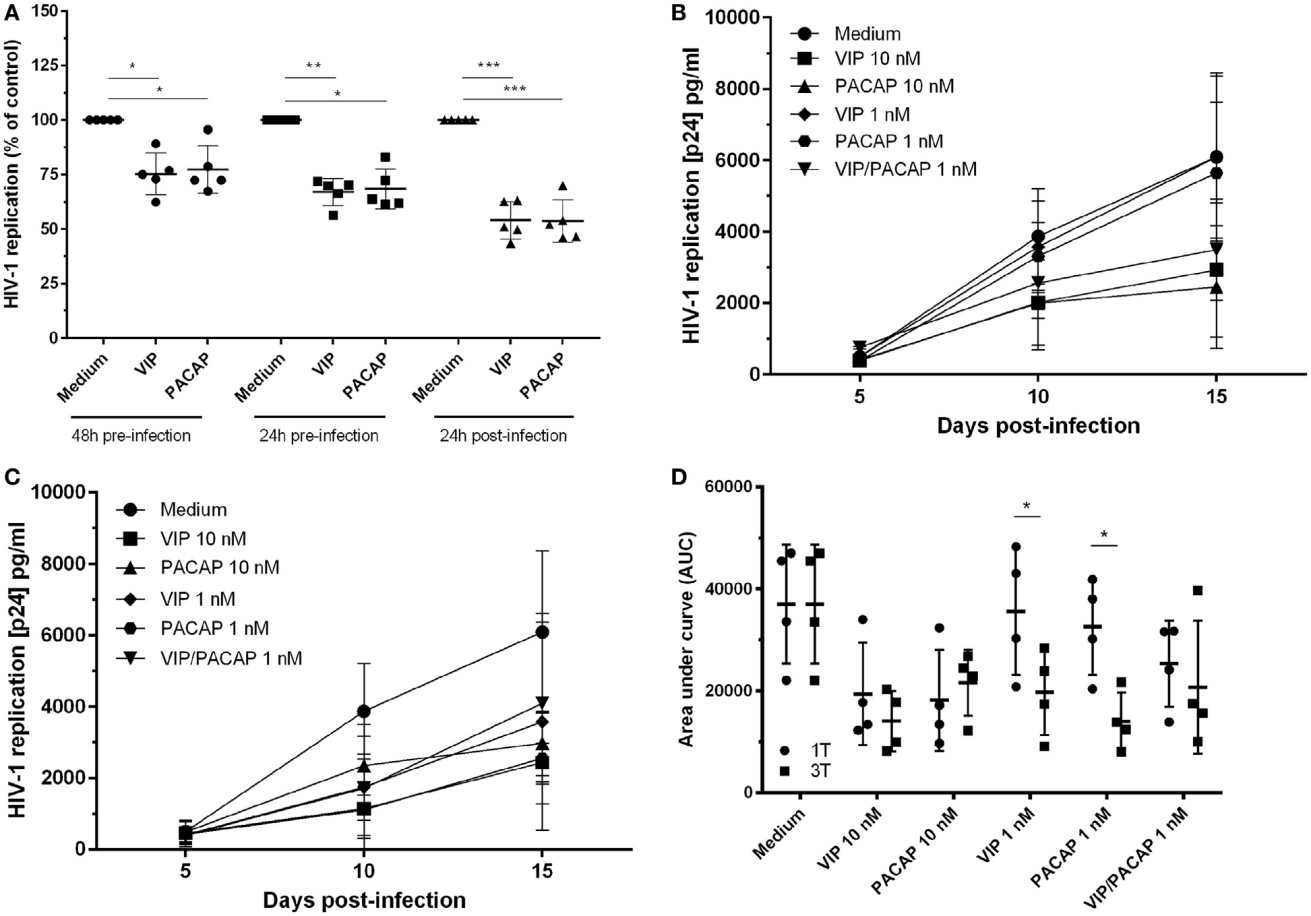
Vasoactive intestinal peptide (VIP) and pituitary adenylate cyclase-activating polypeptide (PACAP) retain the anti-HIV-1 effect at different modes of addition to macrophages. **(A)** Macrophages were treated with VIP or PACAP (10 nM) 24 or 48 h prior, or 24 h after infection. After 14 days of infection, supernatants were collected and viral replication was measured (*n* = 5). Macrophages were infected and treated with different concentrations of VIP or PACAP in a regime of one dose at day 1 [**(B)**, 1T] or three doses at day 1, 5, and 10 [**(C)**, 3T]. Supernatants were collected 5, 10, and 15 days after infection. **(D)** Area under curve (AUC) analysis of panels **(B,C)** (*n* = 4). *p < 0.05; **p < 0.01; ***p < 0.001; two-way ANOVA, with Tukey post-test.

To examine whether both neuropeptides were able to render uninfected macrophages less susceptible to HIV-1 infection, cells were treated with the neuropeptides and then infected. We found that pre-exposure to VIP and PACAP (10 nM) resulted in long-term inhibition of HIV-1 replication, albeit to a lesser extent compared to post-infection treatment (Figure 1A). Following, we compared two treatment protocols: a single post-infection treatment (day 1) using optimal and suboptimal VIP and PACAP concentrations (relative to inhibition of HIV-1 replication) (Figure 1B) and a consecutive treatment with three doses of each neuropeptide (on day 1, 5, and 10) at both concentration levels (Figure 1C). Applying area under curve analysis, we observed that when infected macrophages were exposed to three non-functional doses of VIP or PACAP, HIV-1 replication was reduced to a level similar to that observed when cells were treated with single functional doses (Figure 1D).

### VIP and PACAP Do Not Change the Expression of CD4 and CCR5 in Macrophages

Taking into account our previous findings that HIV-1 inhibition by VIP and PACAP is partially dependent on β-chemokines (27), we tested the possibility that these neuropeptides could directly modulate the expression of CCR5 in macrophages. We observed that the macrophage expression levels of CCR5 and CD4 as well were not changed after cell exposure to either neuropeptide during 24 h (Figures S1A–E,H in Supplementary Material), thus excluding reduced expression of cellular HIV-1 receptors as a mechanism of HIV-1 inhibition promoted by VIP and PACAP. In addition, the treatment with VIP and PACAP did not change the expression of the macrophage marker CD68 (Figures S1F,G in Supplementary Material).

### VIP and PACAP Do Not Modulate the Expression of Their Receptors in HIV-1-Infected Macrophages

We previously demonstrated that, in addition to VIP and PACAP, specific ligands to the VPAC1, VPAC2, and PAC1 receptors also diminished the production of HIV-1 in macrophages (27), thus indicating that these receptors are present in HIV-1-infected macrophages. However, we had not yet directly detected the presence or quantified the levels of these receptors in our model. Here, we determined the expression levels of VPAC1, VPAC2, and PAC1 in HIV-1-infected macrophages and also examined whether VIP and PACAP could modulate the levels of their own receptors in these cells. As expected, macrophages expressed the three receptors and VIP and PACAP did not significantly change the receptor levels; however, we detected that VPAC1 and PAC1 were expressed in only 20%, and VPAC2 in approximately 30% of cells, without any significant differences in the MFI relative to the basal and treatment conditions (Figures S2A–C in Supplementary Material).

### Activation of cAMP Signaling Contributes to VIP and PACAP-Induced HIV-1 Inhibition

**Figure 2:**
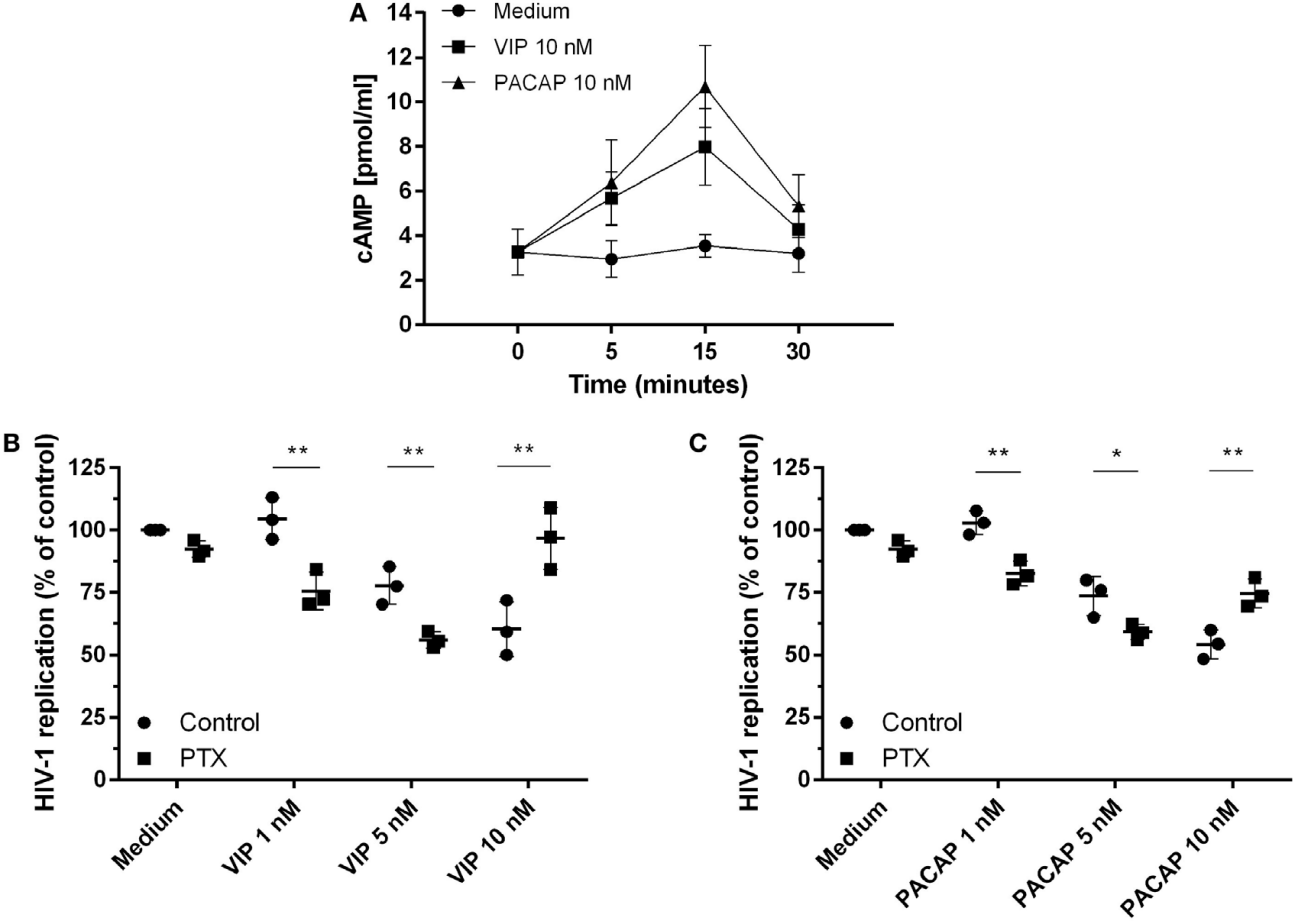
Activation of cAMP signaling contributes to vasoactive intestinal peptide (VIP) and pituitary adenylate cyclase-activating polypeptide (PACAP)-induced HIV-1 inhibition in macrophages. **(A)** Uninfected macrophages were treated with VIP or PACAP (10 nM) and intracellular cAMP levels were analyzed by ELISA at different time points (*n* = 3). **(B,C)** HIV-1-infected macrophages were exposed to pertussis toxin (25 pg/mL) and, 3 h later, cells were washed and treated with VIP or PACAP at different concentrations. Supernatants were collected after 12 days and viral replication was measured (*n* = 3). *p < 0.05; **p < 0.01; ns, not significant; two-way ANOVA, with Tukey post-test.

Because the activation of cAMP signaling has been detected in the majority of VIP and PACAP cell signaling studies (3, 4, 33–35), we analyzed the levels of cAMP production and its role in the HIV-1 inhibitory effect promoted by both neuropeptides. We found that VIP and PACAP raised cellular levels of cAMP, peaking at 15 min of stimulus (Figure 2A), pointing to Gs protein activation by both neuropeptides. Two scenarios are possible regarding cAMP regulation: (1) dependency of the Gs protein only, implying a wide window of tolerance for high levels of cAMP; (2) dependency of both Gs and Gi, inferring a narrow window for the optimal cAMP level, with an increase of cAMP over the maximum point, leading to reversion or blockade of the neuropeptide effect. Using PTX (an inhibitor of Gi protein), we evaluated whether Gi protein blockade (leading to a subsequent extra-amplification of the cAMP levels) could change the VIP and PACAP inhibitory effect on HIV-1 infection, indicating that cAMP pathway activation is a component of the cellular signaling involved in the neuropeptide effects on HIV-1 production in macrophages. However, before using PTX, we searched for a concentration that was able to affect the levels of cAMP without modifying HIV-1 replication, since the B-subunit of PTX (the A-subunit being the Gi inhibitor) is a potent inhibitor of HIV-1 (36). Thus, we reduced the PTX concentration to 25 pg/mL, which preserved the modulation of the cAMP levels, and found that HIV-1 inhibition was negligible (Figures S3A,B in Supplementary Material). In the presence of PTX, an increment in the anti-HIV-1 effect occurred at suboptimal doses of 1 and 5 nM VIP and a complete loss of function was found at the optimal dose of 10 nM (Figure 2B). Concerning PACAP, we observed the same change with the suboptimal doses, but only a partial reduction of the effect with the optimal dose of 10 nM, as HIV-1 inhibition resulting from the combination of PACAP plus PTX was lower than that of viral replication in the presence of the neuropeptide only (Figure 2C). These results indicate that the Gi protein regulates the optimal levels of cAMP that are needed for VIP and PACAP inhibition of HIV-1 replication.

### Effect of VIP and PACAP on HIV-1 Replication Is Dependent on PKA and PKC Activation

**Figure 3:**
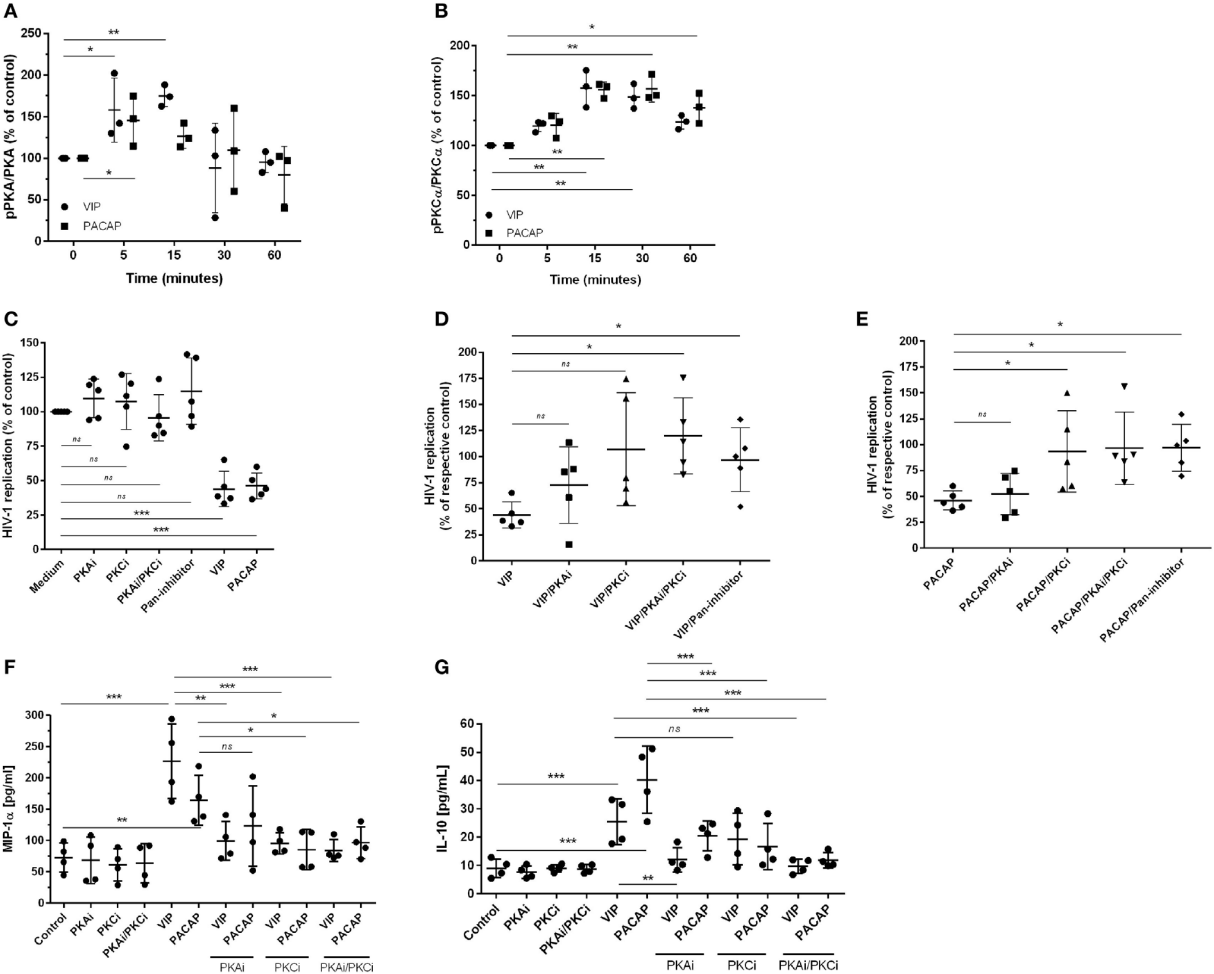
The effect of vasoactive intestinal peptide (VIP) and pituitary adenylate cyclase-activating polypeptide (PACAP) on HIV-1 replication is dependent on protein kinases A (PKA) and C (PKC). **(A,B)** Uninfected macrophages were treated with VIP or PACAP (10 nM) and levels of PKA and pPKA **(A)**, PKCα and pPKCα **(B)** were analyzed by western blot at different time-points; figures show the ratios between band densitometry normalized based on β-actin intensity (*n* = 3). **(C–E)** Infected macrophages were treated with VIP or PACAP (10 nM) in the presence or not of signaling inhibitors (PKAi, H89; PKCi, Gö 6983; Pan-protein kinase inhibitor, H7, 50 nM each one) for 30 min; cells were washed before neuropeptide addition [in **(C)**, results are shown normalized to medium to rule out any possible inhibitor interference on HIV-1 replication]. **(D,E)** Treatment with VIP **(D)** or PACAP **(E)** plus inhibitors; results are shown normalized to respective controls [shown in **(C)**], to compare the individual inhibitory effects. Supernatants were collected after 12 days and viral replication was measured (*n* = 5). **(F,G)** Infected macrophages were treated with VIP or PACAP (10 nM) in the presence or not of signaling inhibitors as above, for 30 min; cells were washed before neuropeptide addition. After 48 h, supernatants were collected and production of MIP-1α **(F)** and IL-10 **(G)** were analyzed by ELISA (*n* = 4). *p < 0.05; **p < 0.01; ***p < 0.001; one-way ANOVA, with Dunnett post-test.

Since PKA and PKC are the main proteins responsible for mediating the physiological actions of VIP and PACAP (3, 4) and because PKA activation occurs secondary to cAMP formation, we analyzed whether the HIV-1 inhibitory effect promoted by both neuropeptides was dependent on the activation of these kinases. We found that VIP and PACAP activated PKA and PKC, as expected, and that the activation of PKA was short-lived (Figure 3A) and activation of PKC was long-lasting and exceeded the analytical time-frame for PACAP (Figure 3B; representative blots are shown in Figure S4 in Supplementary Material). To evaluate the participation of PKA and PKC in the anti-HIV-1 effect of VIP and PACAP, we treated HIV-1-infected macrophages with pharmacological inhibitors of these kinases before exposing them to either neuropeptide. We found that blocking PKA or PKC activation reduced the anti-HIV-1 effect of VIP and PACAP, respectively (Figures 3C–E), whereas blocking both (PKAi plus PKCi), or using a pan-inhibitor (PKA/PKC/PKG), completely disrupted the HIV-1 inhibition promoted by both neuropeptides (Figures 3C–E). We further analyzed whether these signaling events contribute to downstream MIP-1α and IL-10 production, since these molecules participate in VIP- and PACAP-mediated HIV-1 inhibition in macrophages, as we previously described (27). As observed in Figures 3F,G, PKA and PKC are clearly involved in macrophage production of MIP-1α and IL-10 induced by these neuropeptides. More precisely, VIP induction of MIP-1α is dependent of both kinases and that of IL-10 is only dependent on PKA; however, PACAP induction of MIP-1α is only dependent on PKC and that of IL-10 is dependent on both kinases. Of note, all of the pharmacological inhibitors were tested for cellular cytotoxicity; thus, the concentrations used in the assays did not affect macrophage viability (data not shown).

### VIP and PACAP Inhibit NF-kBp65 Phosphorylation in HIV-1-Infected Macrophages

**Figure 4:**
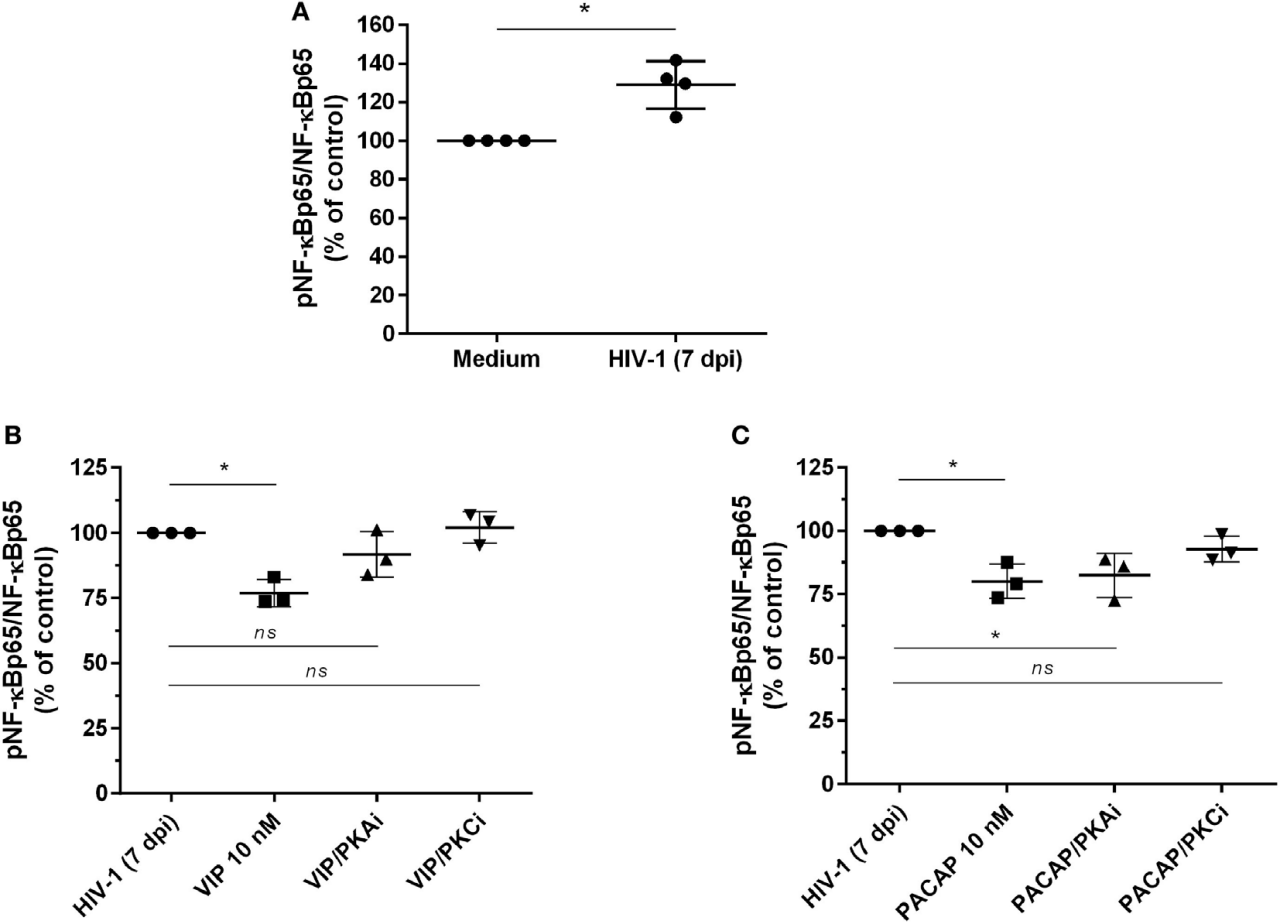
Vasoactive intestinal peptide (VIP) and pituitary adenylate cyclase-activating polypeptide (PACAP) inhibit NF-kBp65 phosphorylation in HIV-1-infected macrophages. **(A)** Macrophages were infected with HIV-1 and 7 days later the levels of NF-kBp65/phosphoNF-kBp65 were analyzed by ELISA (*n* = 4). **(B,C)** Macrophages were treated with VIP or PACAP (10 nM) 7 days after HIV-1 infection (7 dpi), in the presence or not of protein kinases A (PKA) or C (PKC) inhibitors (PKAi, H89; PKCi, Gö 6983; 50 nM each one, for 30 min; cells were washed before neuropeptide addition). After 1 h, the levels of NF-kBp65/phosphoNF-kBp65 were analyzed by ELISA (*n* = 3). *p < 0.05; one-way ANOVA, with Dunnett post-test.

The transcription factor NF-kB is an inherent component of the HIV-1 replicative cycle, and NF-kB inhibition decreases viral production and promotes provirus latency in infected cells (37). VIP and PACAP are inhibitors of NF-kB (20), and this inhibition may contribute to their anti-HIV-1 effect. We thus evaluated the activity of NF-kB in cells exposed to the neuropeptides by detecting the total and phosphorylated NF-kBp65 subunit. To better assess the inhibition phenotype, we first treated uninfected macrophages with TNF-α for 1 h, added VIP or PACAP, and evaluated the levels of NF-kBp65 1 h later. This approach was aimed to promote the activation of NF-kB by TNF-α, which allowed us to analyze whether VIP and PACAP were able to reduce the activation status of NF-kB to basal levels. TNF-α mimics the effect of HIV-1 infection, which activates the NF-kB transcription factor and leads to the production of TNF-α (38, 39). Both neuropeptides reduced TNF-α-mediated activation of NF-kBp65 to levels comparable to those of control cells not exposed to the cytokine, indicating the marked anti-NF-kBp65 activity of VIP and PACAP (Figure S5A in Supplementary Material). We also analyzed the activation of NF-kB using a gene reporter assay in uninfected THP-1 macrophages. The NF-kB reporter construct was induced by TNF-α, but this induction was abrogated when VIP or PACAP was added together with TNF-α (Figure S5B in Supplementary Material). Next, we analyzed the phosphorylation of NF-kB in macrophages infected with HIV-1 for 7 days to achieve a larger virus propagation throughout the culture, thus allowing the increment of basal NF-kB activity by the own HIV-1 infection (Figure 4A) (40, 41). One-hour treatment with VIP and PACAP reduced NF-kBp65 phosphorylation induced by TNF-α (Figures 4B,C). Notably, PKA or PKC blocking prevented VIP inhibition of NF-kB (Figure 4B), whereas only PKC blockage inhibited the effect of PACAP on NF-kB (Figure 4C).

### VIP and PACAP Phosphorylate CREB in HIV-1-Infected Macrophages

**Figure 5:**
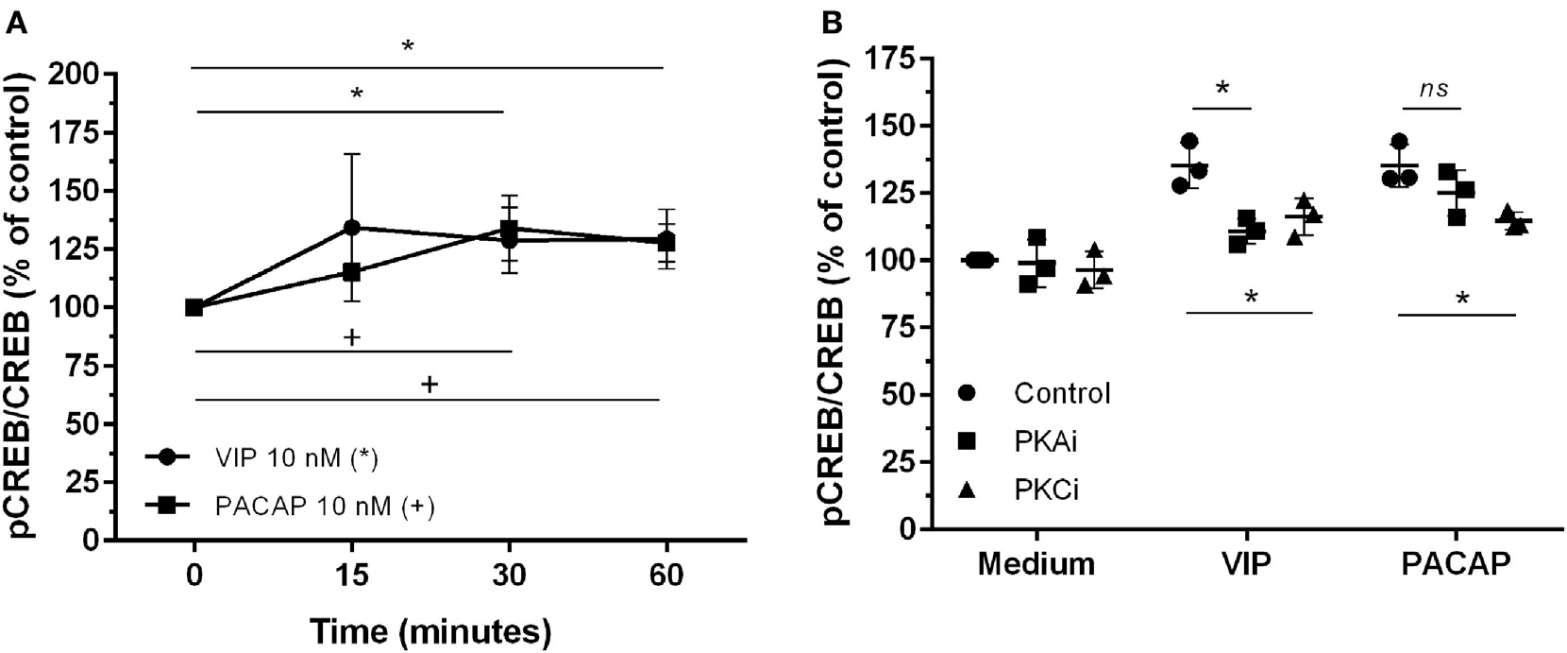
Vasoactive intestinal peptide (VIP) and pituitary adenylate cyclase-activating polypeptide (PACAP) promote cAMP response element-binding protein (CREB) phosphorylation in HIV-1-infected macrophages. **(A)** Uninfected macrophages were treated with VIP or PACAP (10 nM) and levels of CREB and phospho-S133 CREB were analyzed by ELISA at different time-points (*n* = 3). **(B)** Infected macrophages were treated with VIP or PACAP (10 nM) in the presence or not of a protein kinases A (PKA) or C (PKC) inhibitors (PKAi, H89; PKCi, Gö 6983; 50 nM each one, for 30 min; cells were washed before neuropeptide addition), and CREB activation was analyzed 30 min after neuropeptide exposure (*n* = 3). ^*,+^*p* < 0.05; ns, not significant; two-way ANOVA, with Tukey post-test.

The CREB, a classical transcription factor of the cAMP/PKA pathway and Ca^2+^-dependent kinases, is induced by several GPCRs ligands, including VIP and PACAP (42, 43). CREB and NF-kB share the CREB-binding protein/p300 (CBP/p300 protein) as a cofactor, and CREB activation results in the inhibition of NF-kB (44). We observed that VIP and PACAP promoted CREB phosphorylation in uninfected (Figure 5A) as well as in HIV-1-infected macrophages (7 days of infection) (Figure 5B). Under this last condition, PKA or PKC blockage diminished CREB phosphorylation by VIP, while the effect of PACAP on CREB activation was reduced only with PKC blockade (Figure 5B).

### VIP and PACAP Reduce the Levels of Cyclin D1 in HIV-1-Infected Macrophages

**Figure 6:**
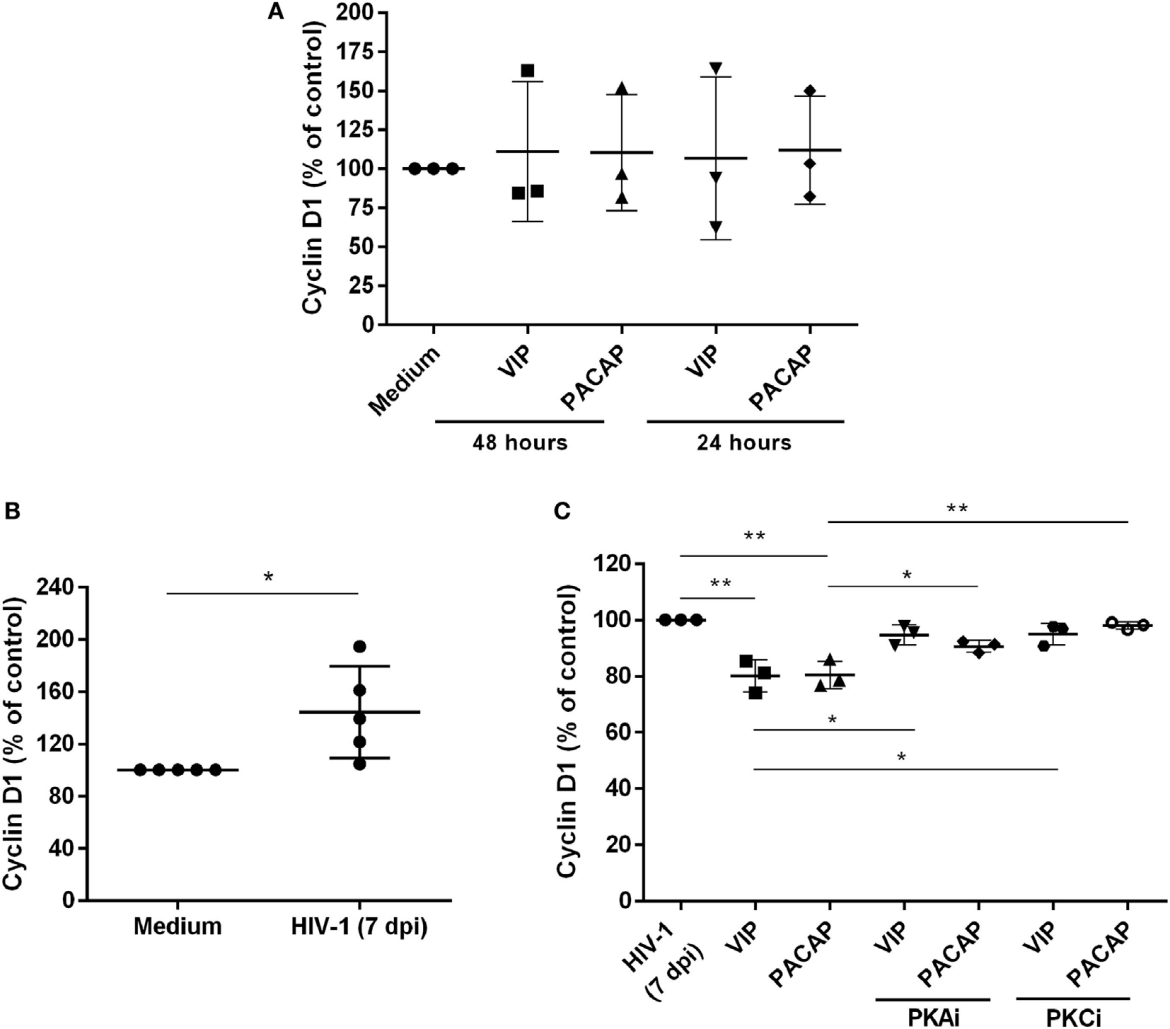
Vasoactive intestinal peptide (VIP) and pituitary adenylate cyclase-activating polypeptide (PACAP) reduce Cyclin D1 levels in HIV-1-infected macrophages. **(A)** Uninfected macrophages were exposed to VIP or PACAP (10 nM) and levels of total Cyclin D1 were quantified by ELISA 24 or 48 h later (*n* = 3). **(B)** Macrophages were infected with HIV-1 and 7 days later the levels of Cyclin D1 were analyzed by ELISA (*n* = 5). **(C)** Macrophages were treated with VIP or PACAP (10 nM) 7 days after HIV-1 infection (7 dpi), in the presence or not of protein kinases A (PKA) or C (PKC) inhibitors (PKAi, H89; PKCi, Gö 6983; 50 nM each one, for 30 min; cells were washed before neuropeptide addition), and levels of Cyclin D1 were quantified by ELISA 24 h later (*n* = 3). *p < 0.05; **p < 0.01; one-way ANOVA, with Dunnett post-test.

We evaluated whether VIP and PACAP could decrease the levels of Cyclin D1, a macrophage protein that is recruited to the HIV-1 transcription complex and participates in viral latency process (45, 46). We initially observed that both neuropeptides did not modulate the Cyclin D1 levels in uninfected macrophages (Figure 6A). On the other hand, we found that HIV-1-infected macrophages (7 days of infection) presented higher expression of Cyclin D1 (Figure 6B) and that both neuropeptides diminished the total levels of this protein in infected cells. In addition, the effects of VIP and PACAP on Cyclin D1 expression were weakened when PKA and PKC activation was blocked (Figure 6C).

### VIP and PACAP Promote Mutations in the HIV-1 Genome

**Figure 7:**
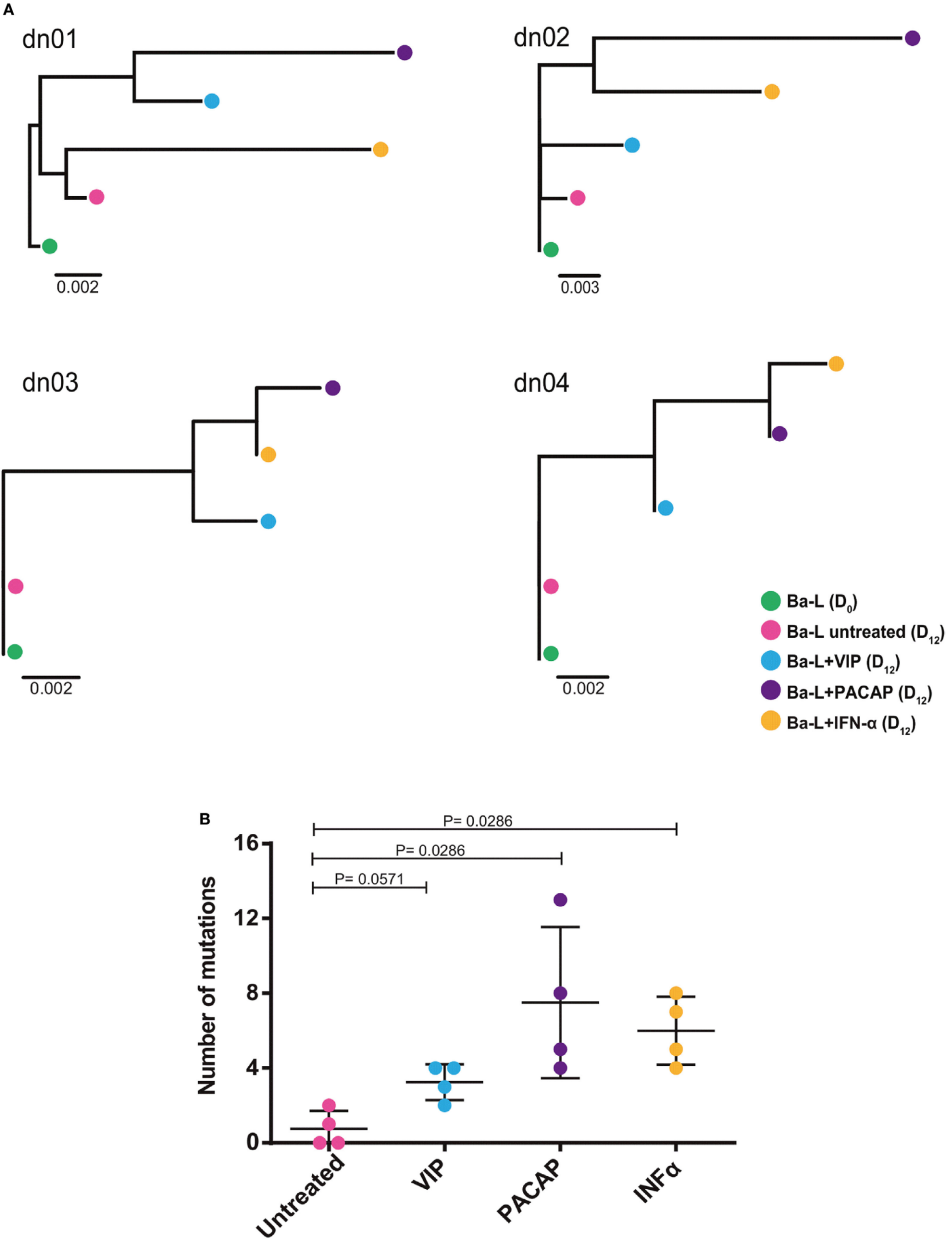
Vasoactive intestinal peptide (VIP) and pituitary adenylate cyclase-activating polypeptide (PACAP) promote mutations in HIV-1 genome. Macrophages from four donor (dn1 to dn4) were infected with the HIV-1 Ba-L isolate (Ba-L[D_0_]) and incubated for 12 days with medium (Ba-L[D_12_]), VIP (10 nM; Ba-L + VIP), PACAP (10 nM; Ba-L + PACAP), or IFN-α (10^3^ U/mL; Ba-L + IFN-α). After that, genomic DNA from macrophages was extracted and the LTR sequences were obtained. **(A)** Neighbor-Joining phylogenetic tree of proviral LTR sequences derived recovered from untreated and treated macrophages. Phylogenetic trees were rooted using the LTR sequence of the original Ba-L(D_0_) isolate. **(B)** Number of mutations respect to the original Ba-L LTR sequence accumulated in proviral genomes recovered from macrophages under different treatment conditions. Horizontal lines indicate the mean and SDs. *p* Values were calculated using the Mann–Whitney test.

Because members of the APOBEC family can be targeted by PKA and PKC (22–24), it is possible that VIP and PACAP modulate these HIV-1 restriction factors, thus promoting mutations in the HIV-1 proviral DNA. To test this hypothesis, infected macrophages were treated with VIP or PACAP [or also IFN-α as a positive control, since it is a potent APOBEC3G inducer (47)], and after 12 days, the mutation profile in the LTR region of the integrated provirus was analyzed. We observed that proviruses obtained from macrophages treated with VIP and PACAP displayed higher genetic distance to the original input (Ba-L[D_0_]) than proviruses from untreated macrophages (Ba-L[D_12_]) (Figure 7A; Table S1 in Supplementary Material). PACAP-treated proviruses accumulated a significantly higher number of mutations than those treated with VIP or untreated proviruses and were comparable to IFN-α-treated proviruses (Figure 7B; Table 1). Notably, a significant proportion of the mutations detected upon treatment with VIP (15%), PACAP (27%), and IFN-α (42%) corresponded to G-to-A substitutions, consistent with mutations derived from the activity of members of the APOBEC family of viral restriction factors (Table 1), and were two, eight, and ten times higher than those detected in control Ba-L(D_12_) proviruses, respectively (Table 1). The complete nucleotide alignment of the HIV-1 LTR sequences is in Figure S6 in Supplementary Material.

**Table 1:** Description of total and specific G to A mutations identified in HIV-1 provirus from infected cells exposed to different treatments.

Treatment	Donor ID	Number of mutations respect to Ba-L	Number of G → A mutations respect to Ba-L
None	dn01	2	1
	dn02	1	0
	dn03	0	0
	dn04	0	0
	Mean	0.75 ± 0.96	0.25 ± 0.50

Vasoactive intestinal peptide	dn01	4	0
	dn02	3	0
	dn03	4	1
	dn04	2	1
	Mean	3.25 ± 0.96	0.50 ± 0.58

Pituitary adenylate cyclase-activating polypeptide	dn01	8	2
	dn02	13	4
	dn03	5	1
	dn04	4	1
	Mean	7.50 ± 4.04	2.00 ± 1.41

INFα	dn01	7	5
	dn02	8	3
	dn03	4	1
	dn04	5	1
	Mean	6.00 ± 1.83	2.50 ± 1.92

### VIP and PACAP Reduce HIV-1 Replication Fitness

**Figure 8:**
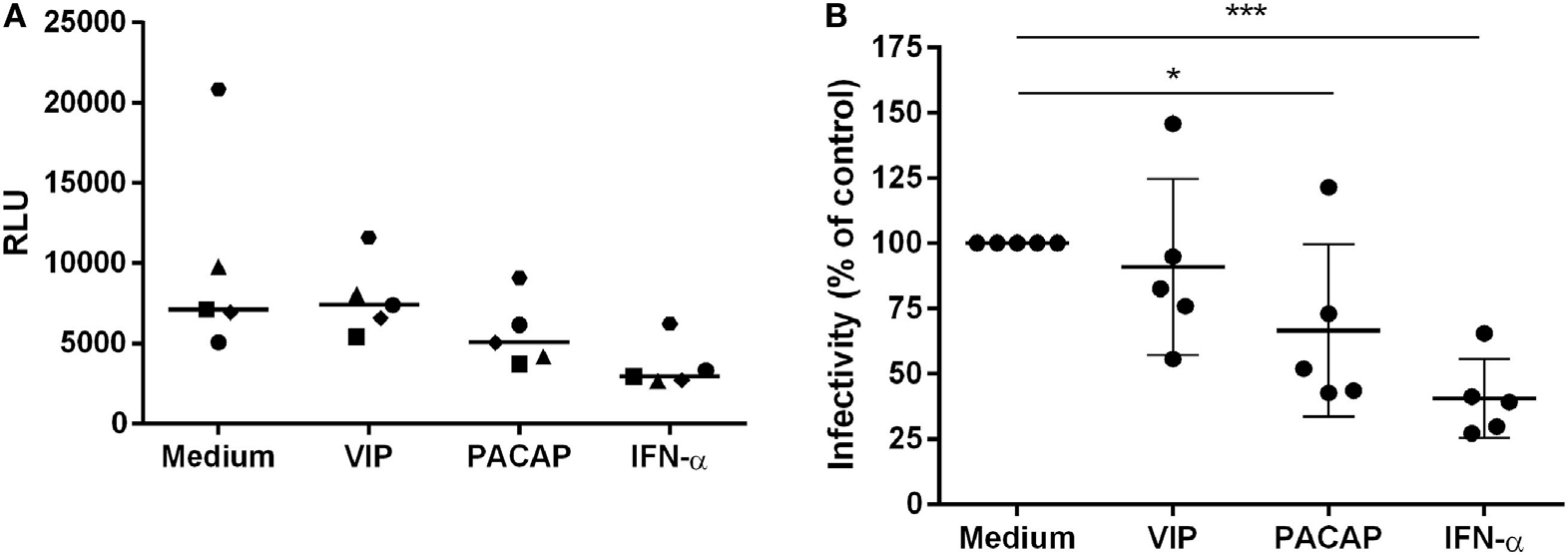
Vasoactive intestinal peptide (VIP) and pituitary adenylate cyclase-activating polypeptide (PACAP) reduce HIV-1 replication fitness. TZM-bl cells were exposed to 5 ng/mL of normalized HIV-1 infectious input, obtained from 12 days-infected macrophages treated with VIP (10 nM), PACAP (10 nM), or IFN (10^3^ U/mL). After 48 h, TZM-bl cells were lysed and luciferase activity was measured. **(A)** Relative luminescence units; bars represent the median of each treatment; **(B)** normalized data from **(A)** (*n* = 5). *p < 0.05; ***p < 0.001; one-way ANOVA, with Dunnett post-test.

To verify the impact of these APOBEC3 signature mutations in viral infectivity, supernatants were collected 12 days after macrophage infection, centrifuged, and clarified. Virions were concentrated, titrated (the same donors as in Figure 7 and an additional donor) and added to TZM-bl cells at normalized amounts to evaluate infectivity. Treatment with VIP did not significantly alter HIV-1 infectivity, whereas viruses derived from PACAP- or IFN-α-treated HIV-1-infected cells significantly lost infectivity (Figures 8A,B). These results suggest that the APOBEC signature mutations introduced into the HIV-1 genome could be related to the ability of PACAP to reduce HIV-1 replication and, consequently, weaken viral propagation in culture. Therefore, we believe that VIP/PACAP-mediated inhibition of HIV-1 infection relies not only on the reduction of virus production but also on the concomitant loss of virus fitness.

## Discussion

Vasoactive intestinal peptide and PACAP, together with other peptides, regulate the neuro-immune-endocrine system and participate in a variety of processes, such as embryogenesis, memory and learning, hormone production, and immune responses. Although both neuropeptides have been extensively studied in many fields, data regarding their actions on specific cell types and influence on infectious processes are still scarce. In this sense, we previously described the ability of VIP and PACAP to limit HIV-1 replication in macrophages and identified some of the mechanisms involved in this activity. Here, we sought to deepen our knowledge of the interaction of VIP and PACAP with human primary macrophages and to search for additional mechanisms contributing to the anti-HIV-1 activity of both neuropeptides.

The variety of ways that these peptides promote HIV-1 replication inhibition suggests that multiple mechanisms may be involved in this phenomenon, in addition to the production of β-chemokines and IL-10 (27). Modulation of transcription factors and viral restriction agents and activation of receptors or the enzymes related to HIV-1 replicative cycle may contribute to the inhibitory phenotype. These mechanisms could act separately or together, preceding those already observed by us or even being redundant, compensating for the absence of each other by virtue of the type of stimulus or cellular state at the moment of interaction with VIP and PACAP. We also detected that VIP and PACAP receptors were expressed in HIV-1-infected macrophages, corroborating the findings of our previous work in which specific agonists for each of the three receptors were shown to have quantifiable effects on HIV-1 replication (27).

Here, we found that VIP and PACAP elicited macrophage cAMP synthesis and PKA and PKC activation, although PKC activity appeared to be predominant over PKA activation following PACAP exposure. Considering studies on other cell types, PACAP is a strong activator of PKA along with PKC (48). For example, PAC1 associations with the Gαs, Gαi, and Gαq subunits are reported to trigger PKA activation, PKA inhibition, and PKC activation, respectively (49). In addition, the PAC1 receptor itself presents several isoforms that have different binding affinity and signaling activity (1). In addition, in several models, the actions of PACAP are related to the activation of the *exchange protein directly activated by cAMP* (EPAC), which directly binds cAMP and exhibits guanine nucleotide exchange factor activity (50). Therefore, it is also possible that, as indicated by our results, the induction of cAMP by PACAP could lead to EPAC activation, generating the differences we observed in the NF-kB and CREB assays. The potential triggering of EPAC signaling pathway raises the possibility that, although HIV-1 replication inhibition by VIP and PACAP is dependent on PKA and PKC activation, other possible inhibitory mechanisms could be differently modulated, depending on the neuropeptide in question.

The difference between VIP and PACAP observed in the experiments using PTX could be explained by the fact that the PACAP receptor PAC1 mainly couples with the Gs and Gq protein subunits (51). Also, the blockade of Gi by PTX could lead to excessive levels of cAMP, promoting receptor desensitization, as described elsewhere (52). These results suggest that VIP action on HIV-1 replication inhibition could more dependent on cAMP signaling than PACAP.

Regarding the analysis of nuclear factors and the final components of the signaling pathways, we observed that VIP and PACAP promoted CREB activation and inhibited NF-kBp65. The investigation of the neuropeptide effects on these two targets was based on the fact that NF-kB is a crucial factor for HIV-1 transcription (37), and on findings reported by others authors indicating that VIP can inhibit the activity of this factor in many cellular models (20, 53, 54). CREB is one of the final components of the signaling promoted by GPCRs that activates PKA and PKC (55, 56). CREB can act as a negative regulator of NF-kB, since both CREB and NF-kB share the accessory protein CBP/p300 and participate in the structure of the transcriptional complex formed by CREB and NF-kB (44, 57). The data we obtained favor the hypothesis regarding the negative regulation of NF-kB by CREB, but we cannot exclude the possibility that VIP- and PACAP-mediated regulation of both transcription factors may be not interconnected.

**Figure 9:**
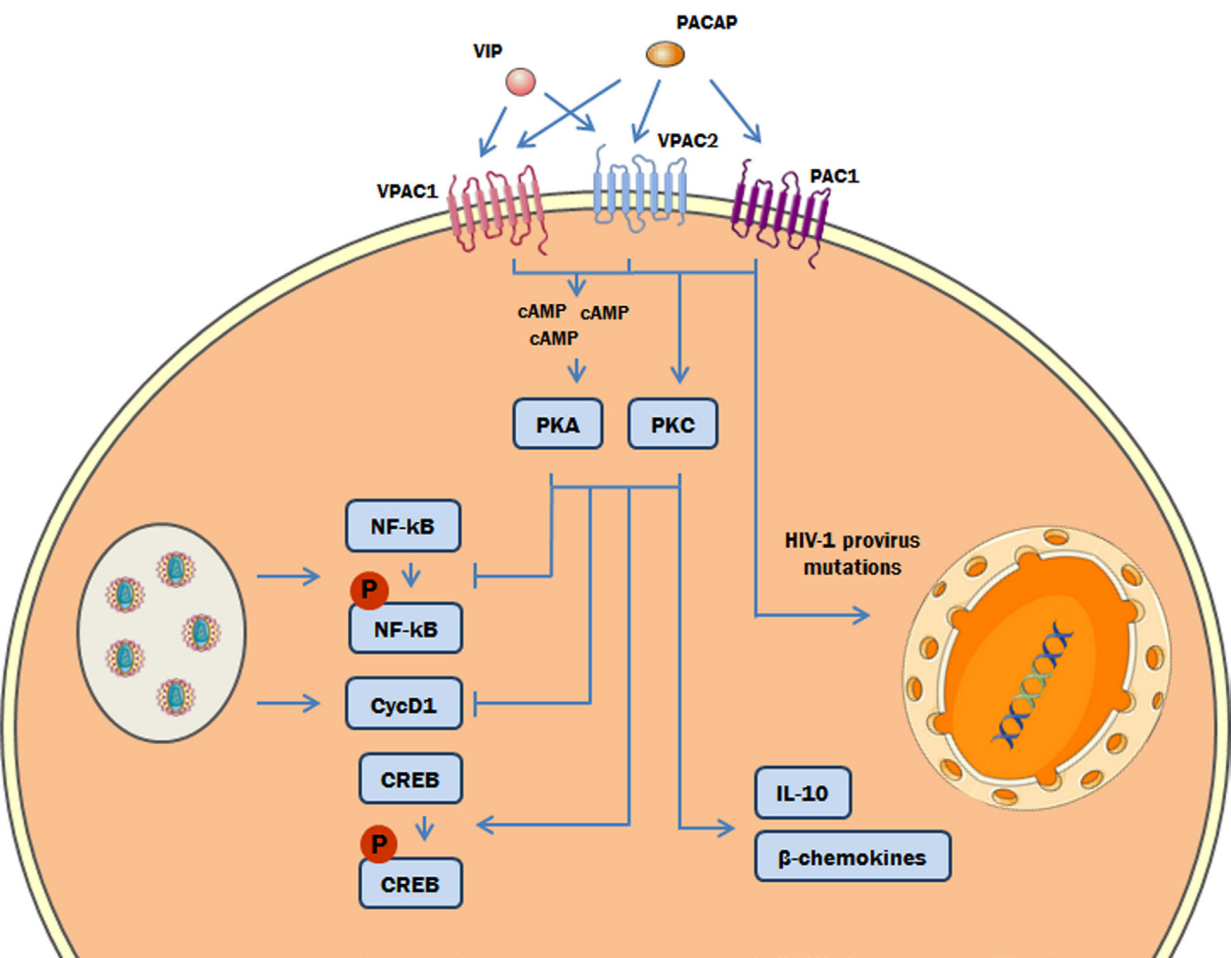
Proposed model of the role of protein kinases A (PKA) and C (PKC) in the vasoactive intestinal peptide (VIP)/pituitary adenylate cyclase-activating polypeptide (PACAP)-mediated inhibition of HIV-1 replication in macrophages. The interaction of VIP and PACAP with their specific receptors in HIV-1-infected macrophages activates PKA (*via* induction of cAMP) and PKC, leading to inhibition of NF-kB activation and Cyclin D1 synthesis induced by HIV-1 infection, and to reduction of HIV-1 production. Also, PKA and PKC activation can trigger the phosphorylation of cAMP response element-binding protein (CREB) and the macrophage production of β-chemokines and IL-10, which diminish HIV-1 replication (27). In addition, PACAP introduces mutations on HIV-1 provirus that result in a reduction of HIV-1 replication fitness (the color green in the DNA represents the integrated HIV-1 provirus).

Overall, our results are internally connected, once they were obtained following the classical signaling pathways triggered by both neuropeptides. Nonetheless, to integrate all of the evidence concerning the cellular signaling cascades involved in the anti-HIV-1 effect of both neuropeptides, it is essential to take into account that the properties of VIP and PACAP to induce HIV-1 inhibitory mediators and regulate transcription factors are both equally dependent of PKA and PKC activation (Figure 9). In this regard, in our previous work (27), we showed that HIV-1 inhibition by VIP and PACAP was dependent on β-chemokines and IL-10 induction; here, we confirmed that the production of these anti-HIV-1 inhibitory factors is also determined by PKA and PKC activation. The transcription factor NF-kB can be modulated in several ways: through regulation of IKK, direct phosphorylation, proteasomal tagging, and competition with its co-activators (58, 59). The participation of PKA in NF-kB inhibition has also been reported by others (60–64), supporting our findings that PKA and PKC blockade prevented the inhibitory effects of VIP and PACAP on NF-kB activation. Likewise, activation of CREB can occur *via* direct phosphorylation by PKA and PKC (65, 66), and in some models, the optimal activation of CREB by PKA is co-dependent on PKC and other Ca^2+^-dependent pathways (67). Regarding VIP and PACAP, NF-kB inhibition and CREB activation by both neuropeptides in immune cells have been demonstrated by other authors (20, 21, 68, 69), thus corroborating our findings. In the same sense, the participation of PKA and PKC in NF-kB and CREB modulation complements those previous reports, as these are the main pathways involved in neuropeptide signaling (3, 4, 70).

Modulation of Cyclin D1 by PKA can be either direct or indirect since there are reports showing the direct phosphorylation of Cyclin D1 sites by PKA and its interaction with the PKA regulatory subunit (71, 72) as well as an indirect effect through PKA modulation of p27/kip1, a CDK/Cyclin inhibitory factor (73). These studies and others show that PKA modulates cyclins at multiple levels. Most studies demonstrate that PKC mainly acts on cyclins indirectly, through inhibition of protein translation (74), modulation of transcription (75), and phosphorylation of p27/kip1 (76). The modulatory effects of VIP and PACAP on the Cyclin D1 levels show that these neuropeptides have great potential to modify viral replication directly in the cellular nuclei through interfering with the transcription of viral proteins and duplication of the genome to form new viral particles. These data allow us to consider that other members of the cyclin group, including CDKs and their inhibitors, can also be regulated by VIP and PACAP. Of note, several of these factors, such as Cyclin L2, Cyclin D3, CDK2, and CDK6, have been described to inhibit the activity of the HIV-1 restriction factor SAMHD1, thus favoring viral replication in macrophages (77, 78).

Another significant finding of the present study concerns the ability of PACAP to induce mutations in the HIV-1 LTR at levels comparable to those of IFN-α. In addition, some of these mutations carry the G-to-A APOBEC signature, and virus progeny from cultures exposed to PACAP have diminished replication fitness, which is also comparable to that induced by IFN-α. The mechanism responsible for these mutations could be a direct consequence of PACAP signaling as PKA and PKC can regulate the expression and activity of APOBEC family members and accessory factors (22–24). Of note, because we only analyzed the LTR region and because the hotspots for mutations are distributed in the entire HIV-1 genome (79, 80), we expect that the mutation profile induced by VIP and PACAP represents a portion of a similar phenomenon that may have occurred in the HIV-1 genome as a whole. The dichotomy between VIP and PACAP in their ability to promote mutations could be explained by the minor differences in the signaling pathways activated by each neuropeptide and/or the induction of final products downstream of the VIP or PACAP pathways, such as their own IFN-α or related factors. These possibilities are currently under investigation in our laboratory.

## Ethics Statement

All experimental procedures involving human cells were approved by the Research Ethics Committee of the Oswaldo Cruz Foundation/FIOCRUZ (Rio de Janeiro, RJ, Brazil) under the protocol number 397-07. All donors provided written consent.

## Author Contributions

Conceived the study: JT, VC, GB, and DB-H. Designed the experiments: JT, SA, DI, RV, PF, VC, GB, and DB-H. Performed the experiments: JT, SA, DI, RV, and PF. Analyzed the data: JT, SA, DI, RV, PF, VC, GB, and DB-H. Wrote the paper: JT and DB-H. All authors reviewed the manuscript.

## Conflict of Interest Statement

The authors declare that the research was conducted in the absence of any commercial or financial relationships that could be construed as a potential conflict of interest.
