# Supplementary material for: The Neuropeptides Vasoactive Intestinal Peptide and Pituitary Adenylate Cyclase-Activating Polypeptide Control HIV-1 Infection in Macrophages Through Activation of Protein Kinases A and C
Source: Front Immunol. 2018 Jun 12;9:1336. doi: 10.3389/fimmu.2018.01336 (PMC6008521; doi:10.3389/fimmu.2018.01336)
Supplement: Supplementary file 1 [file Data_Sheet_1.PDF]

Supplementary Material

The neuropeptides VIP and PACAP control HIV-1 infection in macrophages through activation of protein kinases A and C.

Jairo R. Temerozo[1,4*], Suwellen S.D. de Azevedo[2], Daniella B. R. Insuela[3], Rhaissa C. Vieira[1], Pedro L.C. Ferreira[1], Vinícius F. Carvalho[3,4], Gonzalo Bello[2], Dumith Chequer Bou-Habib[1,4*].

[*]**Correspondence:** Dumith Chequer Bou-Habib: dumith@ioc.fiocruz.br *or* dumith.chequer@gmail.com; and Jairo R. Temerozo: jairojrt@gmail.com

1 Supplementary Data

1.1 Cellular expression of CD4, CCR5 and VIP/PACAP receptors

Macrophages cultured in 6-well plates were washed in ice-cold PBS, detached from culture dishes using cell-scraper, and then incubated during 15 minutes in blocking solution containing 10% normal human and mouse serum, and 1% FcBlock solution (eBiosciences, USA) in PBS. Cells were stained with mouse anti-CD4-PE and mouse anti-CCR5-APC-Cy7 (BD Bioscience, USA), suspended in blocking buffer for 20 minutes at room temperature, and then permeabilized (Intracellular Fixation & Permeabilization Buffer Set; Thermo Fisher, USA). Next, cells were stained with mouse anti-CD68-Alexa 647, washed and fixed. For VIP and PACAP receptors analysis, macrophages were permeabilized after blocking, and exposed to rabbit anti-VPAC1, anti-VPAC2 or anti-PAC1 monoclonal antibodies (Abcam, USA) for 20 minutes. After washing, cells were stained with goat anti-rabbit-Alexa 546 and mouse anti-CD68-Alexa 647 for 20 minutes, washed and fixed. Data were acquired with a BD Canto II flow cytometer using BD FACSDiva software (BD Bioscience, USA). Automatic compensation was performed at the beginning of each experiment and

“fluorescence minus-one” was used for gate plotting. Data were analyzed using FlowJo v10 (TreeStar Software, USA).

1.2 Cytotoxicity assay

To test the safety of the pharmacological inhibitors to human macrophages, these cells were exposed to the compounds at their usage concentrations for 72 hours, in 96-well plates. Then, 50 µL of a 1 mg/mL solution of 2,3-Bis(2-methoxy-4-nitro-5-sulfophenyl)-2H-tetrazolium-5-carboxanilide inner salt (XTT; Sigma, USA) with N-methylphenazonium methyl sulfate (PMS; Sigma, USA) dissolved in DMEM without serum, were added to the cell culture. After 3–4 hours, the optical density (OD) was measured using an automatic plate reader with 450 nm test and 690 nm reference wavelengths.

2 Supplementary Figures and Tables

2.1 Supplementary Figures

Supplementary Figure 1

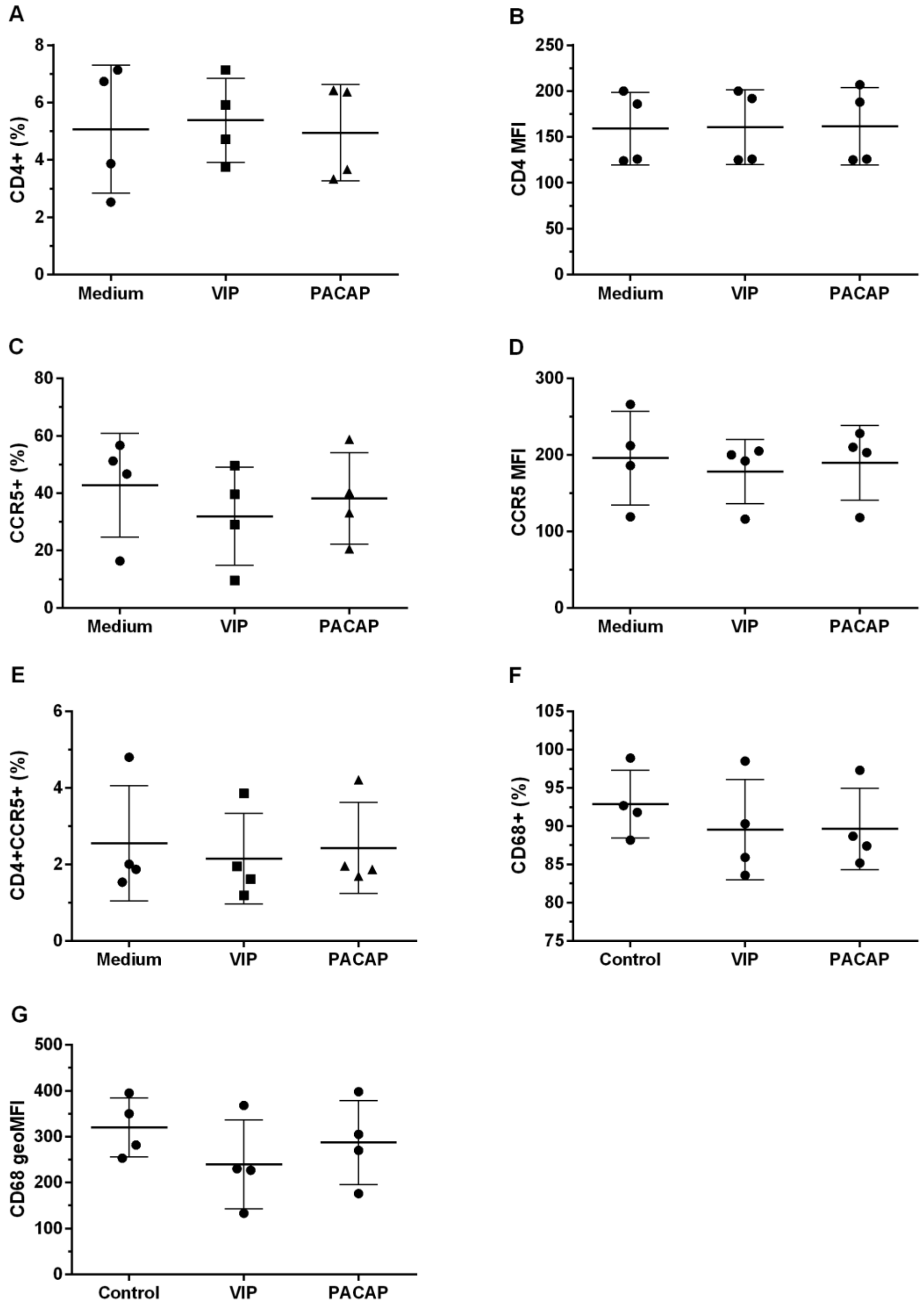

H

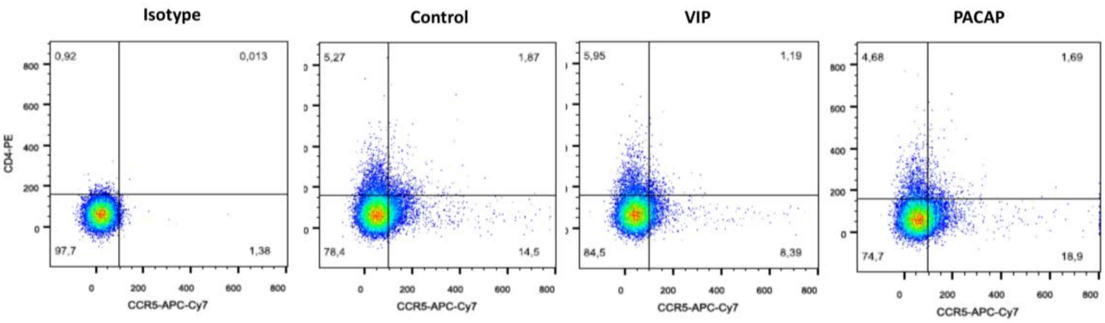

Supp. Figure 1: VIP and PACAP do not change the expression of CD4, CCR5 and CD68 in macrophages. Macrophages were treated with VIP or PACAP (10 nM) and the expression of CD4, CCR5 and CD68 were analyzed by flow cytometry 24 hours later. (A, C, E and F), Analysis of frequency of indicated cell populations; (B and D), Analysis of mean or (G) geometric mean fluorescence intensity (n=4). (H) Representative plot of the results shown from A-E.

Supplementary Figure 2

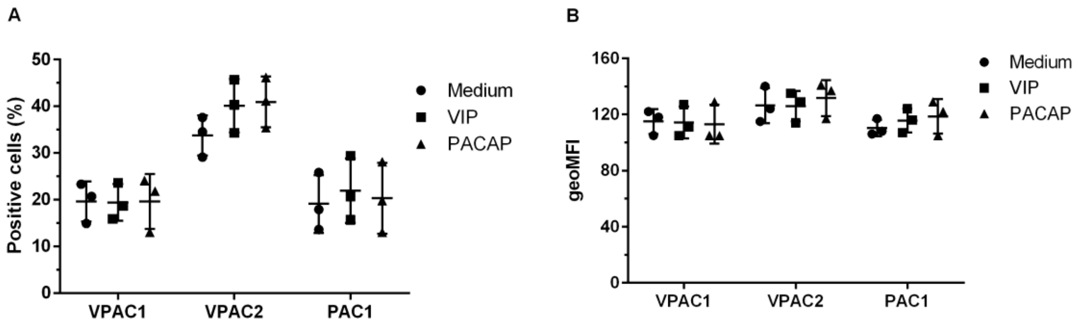

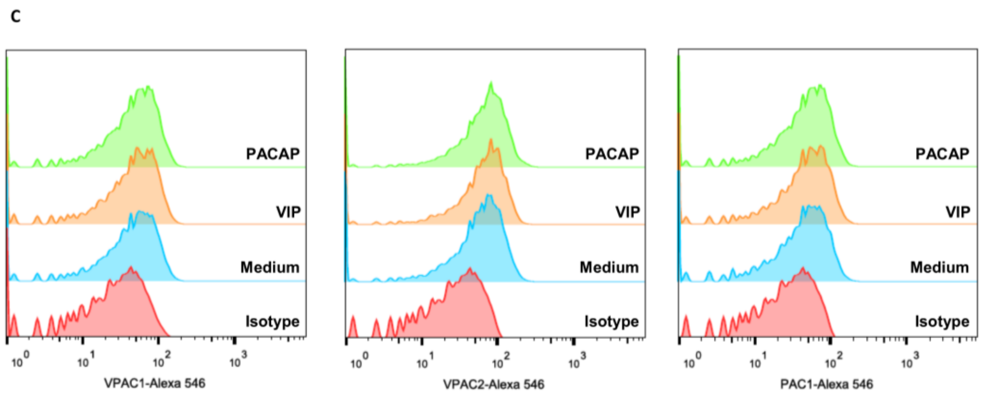

Supp. Figure 2: VIP and PACAP do not modulate their receptors in HIV-1-infected macrophages. Infected macrophages were treated with VIP or PACAP (10nM) and expression of VPAC1, VPAC2 and PAC1 were analyzed by flow cytometry 24h later. (A), Analysis of frequency of positive cells; (B), Analysis of geometric mean fluorescence intensity (n=3). (C), Representative histogram of the results shown in B.

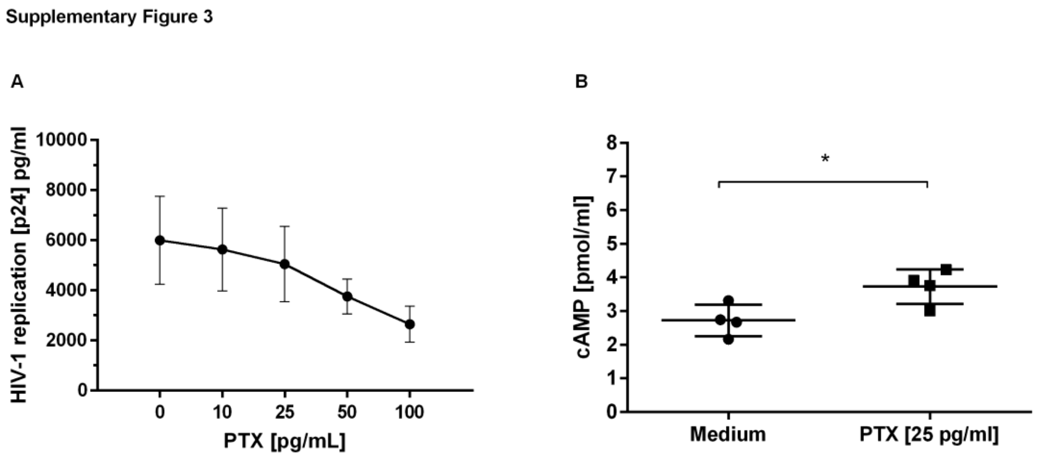

Supp. Figure 3: Detection of PTX sub-optimal concentration for HIV-1 replication. (A) HIV-1-infected macrophages were exposed to different concentrations of PTX; after 3 hours, cells were washed and maintained in culture for 12 days, when supernatants were collected and the viral replication was measured (n=3). (B) Uninfected macrophages were treated with PTX (25

ng/ml) in the presence of 500 nM of IBMX (a competitive nonselective phosphodiesterase inhibitor). After 3 hours, the intracellular levels of cAMP were analyzed by ELISA (n=4). *, $p<0.05$, One-way ANOVA, with Dunnett post-test.

Supplementary Figure S4

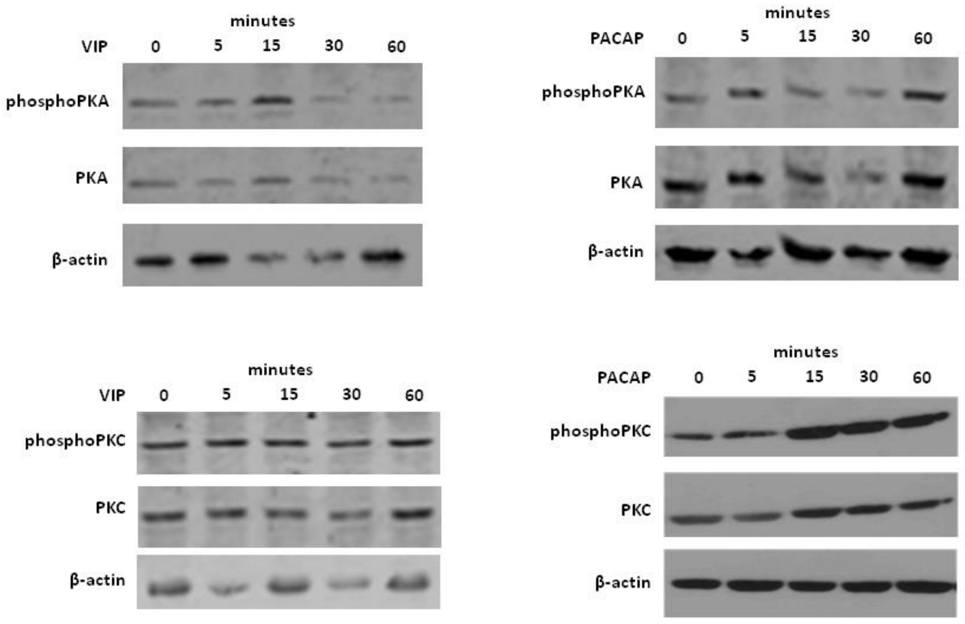

Supp. Figure 4: Representative blot of kinase activation. Macrophages were treated with VIP or PACAP (10 nM) for different time-points and then PKA or PKC activation was analyzed by standard immunoblotting (See Materials and Methods section). Protein expression was detected using enhanced chemiluminescence (see details in Materials and Methods section).

Supplementary Figure 5

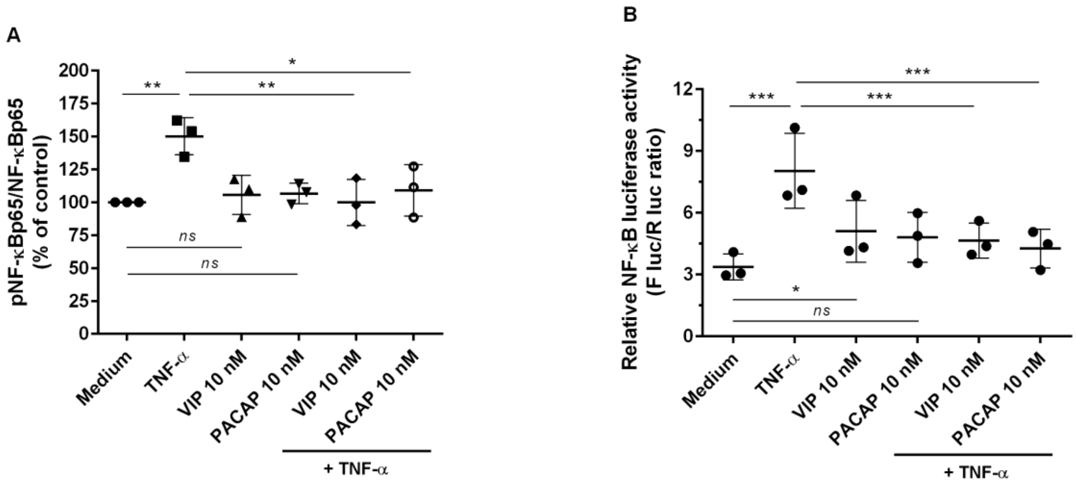

Supp. Figure 5: NF-kB analysis in uninfected macrophages and THP-1 cells. (A) Uninfected primary macrophages or (B) THP-1 macrophages transiently transfected with the 6kB-Luciferase consensus vector were treated with TNFα (10 ng/ml) and, after 1 hour, were exposed to VIP or PACAP (10 nM). After 1h, the ratio between NF-kBp65/phosphoNF-kBp65 was quantified by ELISA (A) and NF-kB transcriptional activity was evaluated by Luciferase/Renilla assay (B). (n=3) ns, no significance; *, $p<0.05$; **, $p<0.01$; ***, $p<0.001$; One-way ANOVA, with Dunnett post-test.

Supplementary Figure 6

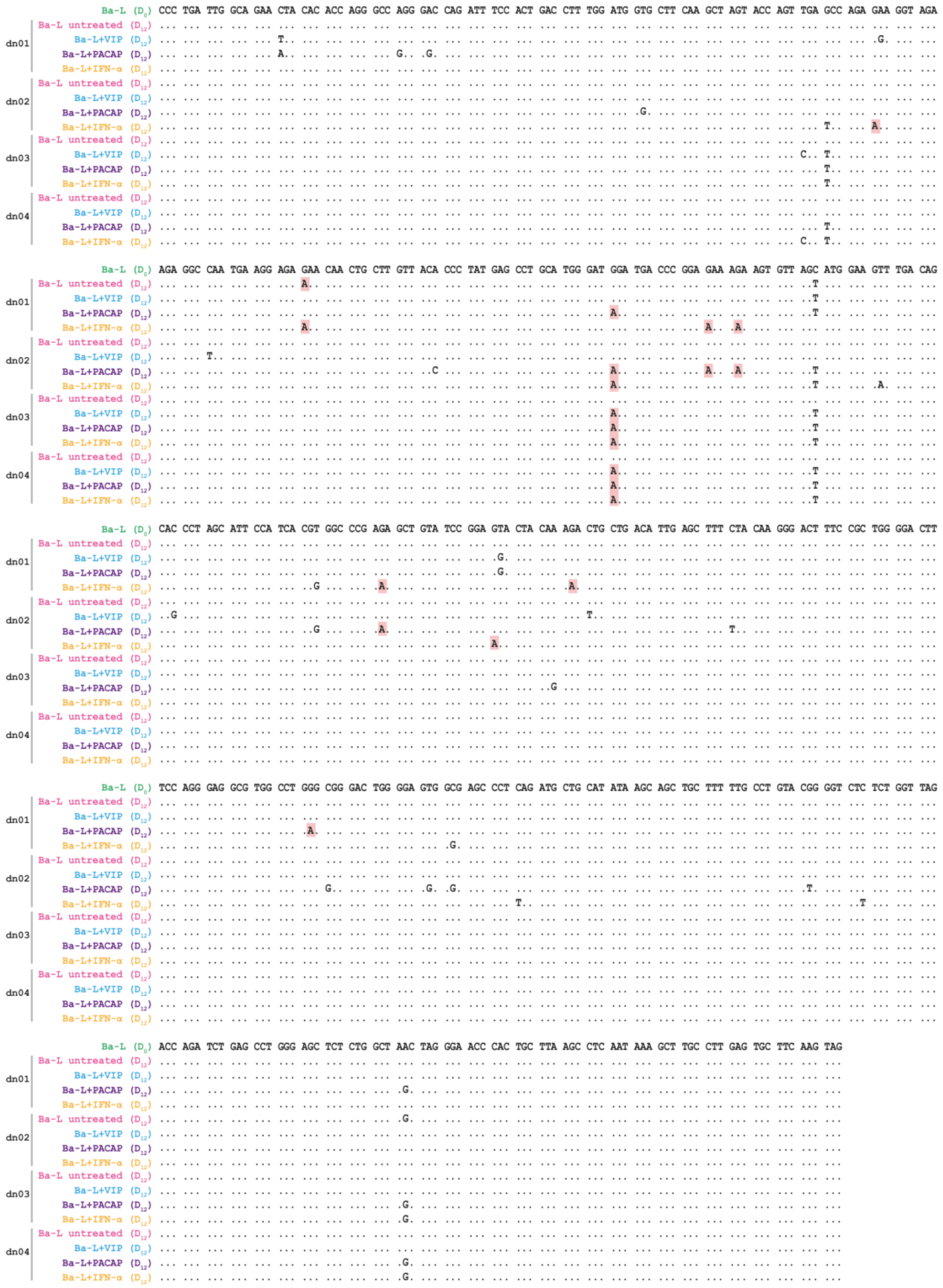

Supp. Figure 6: HIV-1 LTR nucleotide sequence alignment. Nucleotide sequences were aligned using ClustalW implemented in MEGA 7 program (71) and then manually edited, yielding a final alignment covering positions 57–580

relative to the HXB2 reference genome. Neighbor-Joining (NJ) phylogenetic trees were reconstructed under the Tamura-Nei nucleotide substitution model (72) using Mega v7 program (n=4).

2.2 Supplementary Table

Table S1

Subject ID	Treatment	Mean genetic distance to Ba-L
dn01	None	0.7%
	VIP	1.0%
	PACAP	1.7%
	INFα	2.4%
dn02	None	0.2%
	VIP	0.6%
	PACAP	2.7%
	INFα	1.7%
dn03	None	0%
	VIP	0.9%
	PACAP	1.1%
	INFα	0.9%
dn04	None	0%
	VIP	0.5%
	PACAP	0.9%
	INFα	1.2%

Table S1: Description of the mean genetic distance to Ba-L (D_0) of HIV-1 provirus from infected cells exposed to different treatments.
